# Effects of Temperature and Relative Humidity on the Embryonic Development of *Hypera postica* Gyllenhal (Col.: Curculionidae)

**DOI:** 10.3390/insects12030250

**Published:** 2021-03-16

**Authors:** Alexandre Levi-Mourao, Filipe Madeira, Roberto Meseguer, Addy García, Xavier Pons

**Affiliations:** 1 Department of Crop Protection and Forest Sciences, University of Lleida, 25198 Lleida, Spain; alexandrelevi.garcia@udl.cat (A.L.-M.); roberto.meseguer@udl.cat (R.M.); addy.garcia@udl.cat (A.G.); 2 MORE CoLab. Mountains of Research Collaborative Laboratory, Environmental and Ecosystem Management, 5300-358 Bragança, Portugal; fmadeira@morecolab.pt

**Keywords:** alfalfa weevil, egg, survival, rate of development, oviposition period, phenology

## Abstract

**Simple Summary:**

The alfalfa weevil, *Hypera postica*, is a destructive pest around the world and has become, in recent years, the most important pest of alfalfa in Spain. The damage from these weevils reduces the alfalfa forage yield and quality. The factors that affect the population development are poorly known in Europe. This study integrates laboratory experiments and field sampling to address population growth at different temperature and humidity conditions. In the laboratory experiments, we evaluated the combined effect of eight temperatures (8, 12, 16, 20, 24, 28, 32, and 36 °C) and three relative humidity regimes (low, medium, and high) on the survival and development time of eggs of Spanish pest populations. The lowest egg survival occurred at high temperatures and low relative humidity. The egg developmental time decreased from 8 to 32 °C, and, at low relative humidity (RH), this time was longer. The minimum threshold and thermal requirements for egg development were determined. In the field sampling, we obtained information regarding the occurrence of eggs and larvae (in winter) and the adult reproductive status (in autumn). Combining the laboratory and field results, the oviposition period was determined. The results of the study contribute to a better understanding of the annual cycle of *H. postica* in Spain and Europe and are useful for developing sustainable pest management strategies.

**Abstract:**

The combined effect of the temperature and relative humidity on the survival and development time of the eggs of Spanish populations of the weevil *Hypera postica*, a key pest of alfalfa around the world, was evaluated under laboratory conditions. The experimental temperatures ranged from 8 to 36 °C, in 4 °C increments. Three relative humidity ranges were defined: high, medium, and low. Eggs of the alfalfa weevil successfully developed until larval emergence at all of the 24 conditions tested. However, the temperature and relative humidity affected the survival of the eggs. The egg developmental time decreased as the temperature increased from 8 to 32 °C, and the longest time was recorded at a low relative humidity (RH). The relationship between the development rate and temperature fit well to the lineal model for relative humidity. The minimum development threshold (T_0_) and the thermal requirement for egg development (K) ranged between 3 and 4 °C and 209 and 246 degree-days, respectively. According to these values and the occurrence of eggs and larvae (in winter) and adults (in autumn) in field samplings, the period of oviposition was determined. The results of the study contribute to better understanding the annual cycle and phenology of *H. postica* in the Iberian Peninsula and southern Europe.

## 1. Introduction

The alfalfa weevil, *Hypera postica* (Gyllenhal) (Coleoptera: Curculionidae) is a very destructive pest of alfalfa, *Medicago sativa* L., which is the world’s most valuable cultivated forage crop. According to the European literature, females lay eggs in clusters inside the alfalfa stems at the end of autumn and winter [1]. The damage, produced by larvae, consists of defoliation reducing the yield and quality of the forage and causing economic losses in many regions of the world [2,3,4], including the Ebro Valley (north-eastern Iberian Peninsula) [5], where 15–20% of the alfalfa in the Mediterranean region is cultivated. The alfalfa weevil has increased its pest status in the last years and is currently the k-pest of alfalfa in this region [6]. However, the reasons for this increase are still unknown. Although a potential development of insecticide resistance has been suggested [7], this has not been demonstrated, and other factors, such as crop management or environmental conditions, could contribute. The present study is focused on this last aspect.

Despite its importance as an alfalfa pest, the information on the biology, life cycle, and ecology and, therefore, the understanding on how the abiotic environment may affect the alfalfa weevil in Europe is scarce. Temperature is a critical abiotic factor that directly influences the insect life processes of survival, development, reproduction, and biological activities and, hence, their population dynamics [8,9]. Temperature is likely the most important environmental variable affecting embryonic development [10]. The eggs of most coleopteran species develop within a range of temperatures with upper and lower thresholds, outside of which, development is retarded or inhibited [11,12,13,14]. 

The available environmental humidity is another important requirement for egg development [15]. The eggs of some species can obtain sufficient water from the moisture of the air, whereas those of other species must be in contact with water for development [10]. Eggs kept under too dry of conditions may fail to hatch: in some cases, because the embryo within is desiccated; in others, because the chorion itself becomes too hard for the young insect to escape [15]. In many coleopteran insects, egg development is delayed by low humidity in the surrounding air, presumably due to the lack of water in it [16]. However, temperature and water availability may interact, modulating their single effects [17]. 

Most of the studies regarding the temperature-dependent development of *H. postica* have been focused in larvae and pupae and have been carried out with populations from North America and Middle East Asia [14,18]. The results of these studies suggested wide variations in geographical regions [19,20,21]. On the other hand, literature dealing with the effect of abiotic factors on embryonic development is scarce. How successfully and quickly eggs develop will determine the start of the damage period and the quantity of damage. A period of warm weather during the beginning of oviposition results in a very large number of eggs laid in a short time. However, if the temperatures are mild, the laying period can be extended for much of autumn and winter [22,23].

Within the framework of a wider study targeted to determine the effect of the environmental abiotic factors on the development of *H. postica* populations and to predict the occurrence and phenology in southern Europe, the present study aimed to evaluate the combined effects of the temperature and relative humidity on the survival and developmental time of eggs. To do that, we submitted egg batches to different constant temperatures and to three ranges of relative humidity. Field samplings were also performed to record the occurrence of adults in autumn and the winter egg phenology to determine the reproductive adult period and validate the predictions made from the laboratory results.

## 2. Materials and Methods

### 2.1. Laboratory Tests

Constant temperatures were established and maintained in separated cabinets with an 8:16 L:D photoperiod. The experimental temperatures ranged from 8 to 36 °C, in 4 °C increments. Three relative humidity (RH) ranges of the air in the experimental rearing cages were defined: high: 90% < RH < 100%; medium: 50% < RH < 75%; and low: 10% < RH < 35%. The predetermined RH was maintained by the use of agar-agar in high range; with only the biological material covered with mousseline in the medium range; and silica gel 2.5–6 mm with indicator (without cobalt chloride) PA-ACS (PANREAC Barcelona, Spain) in the low range. The temperature and RH were measured using a HOBO^®^ Pro v2 logger U23-00x (ONSET, Cape Cod Massachusetts, MA, USA) probe at the level of the rearing boxes and recorded every 10 min during the experimental period. 

Eggs from *H. postica* were obtained by rearing North-East Spain field-collected adults in 2000 mL glass jars, covered with mousseline for proper ventilation. For egg laying, fresh alfalfa stems were provided on a daily basis. Stems were placed into a filled water glass vial sealed with parafilm to prevent dehydration. Then, the 24 h-old stems were removed and dissected for egg counting. Therefore, the experiment was set with eggs laid in the preceding 24 h. Stems with clusters of 10–15 eggs were placed in transparent PVC rearing cages (53 mm diameter to 32 mm high), and 20 replications for every temperature/RH combination were established. 

The cages were examined at daily intervals, and the number of living eggs and egg colour (distinguishing between yellow, green-brown, and head capsule visible or black (adapted from [23]) was recorded until hatching. As the number of eggs in each cluster varied, the proportion of the finally hatched eggs related to the initial number of eggs was calculated for each replication. We considered that an egg was hatched when the larva was able to break the chorion and leave the eggshell. 

### 2.2. Field Sampling

Twenty-five fields with similar crop conditions were sampled between November and February in 2018–2019 and 2019–2020 to obtain data on egg occurrence and development. The fields were located along four different areas of the Ebro-Valley region (Urgell, Segrià, Baja Cinca, and Monegros) covering a total distance of 150 km in a straight line. Each field was divided into four sectors, and 25 alfalfa stems per sector were picked up, gently excising them at the plant crown level. The stems were brought to the laboratory, kept in a refrigerator at 5 °C and processed within the following 5 days. The stems were dissected using a scalpel to expose the egg clusters. The number of larvae, eggs, and their external colour (yellow, brown, and head capsule visible or black) were recorded.

In addition, adults were collected by sweep-netting in certain fields of each of the four different areas over six weeks, from the beginning of October until middle November. These samples were brought to the laboratory, sexed, and dissected to evaluate their reproductive developmental stage (the presence or absence of eggs in females and sperm in seminal vesicles in males).

### 2.3. Data Analysis

For each temperature and RH combination, we calculated: (1) the age-specific survival rate (l*x*: the probability at birth of being alive at age *x*). The Kaplan–Meier method was used to estimate survival curves for the embryonic stages at each examined temperature and relative humidity. Log-rank tests were used to compare the overall survival curves as well as to compare the survival curves between the relative humidities at each temperature. The R packages used were ‘survival’ [24] and ‘survminer’ [25].

In the analysis of the development time and development rates, only individuals with complete development were included. The effect of the temperature, relative humidity, external egg colour, and their interactions on the duration time was analysed using generalized linear models (GLM) based on a Poisson distribution with a log link function. The Tukey HSD method was used for post hoc comparisons with the ‘agricolae’ package in R [26].

The rate of the embryonic development was also calculated for each temperature and RH level. This rate was used to describe the temperature-dependent development using a linear model [27]. The linear model was easy to build and allows the calculation of the lower development threshold and thermal constant within a range of temperatures. 

Then, taking into account weekly periods from October to February, the average week temperature of the last fifteen years from the four different areas of the Ebro Valley (Urgell, Segrià, Baja Cinca, and Monegros), and the lower temperature threshold, we calculated the daily thermal accumulation (degree-days or DD) for each of the three levels of RH according to the temperature mean method. No upper development threshold was taken into account. The data of the daily temperatures were obtained from the Spanish Agency of Meteorology (AEMET) [28] from the closest meteorological stations to the sampled fields. This procedure allowed us to forecast a theoretical date of egg hatching in each of the four areas of the Ebro Valley and RH regimes.

The first field record of larvae in the winters of 2018–2019 and 2019–2020 was used to determine the beginning of the oviposition period (oviposition window in advance), considering the larval developmental time at 8 °C (the closest temperature to the winter average temperatures) obtained in another experiment dealing with the effect of temperature on the postembryonic development of *H. postica* [29]. In the absence of larvae but with the presence of eggs with visible head capsule, the estimation of the date of egg laying was less accurate but also acceptable. However, the occurrence of adults in autumn and their reproductive developmental stage were used to estimate the actual beginning of the alfalfa weevil egg-laying and were used as a validation of the theoretical prediction of the oviposition starting time. By knowing the duration of the last yellow cluster found at 8 °C, the closest temperature to the winter average temperatures, it was possible to determine the end of the oviposition window (Supplementary Figure S1).

All statistical analyses were performed using R version 3.5.2 (R Foundation for Statistical Computing, Vienna, Austria). 

## 3. Results

### 3.1. Survival

**Figure 1:**
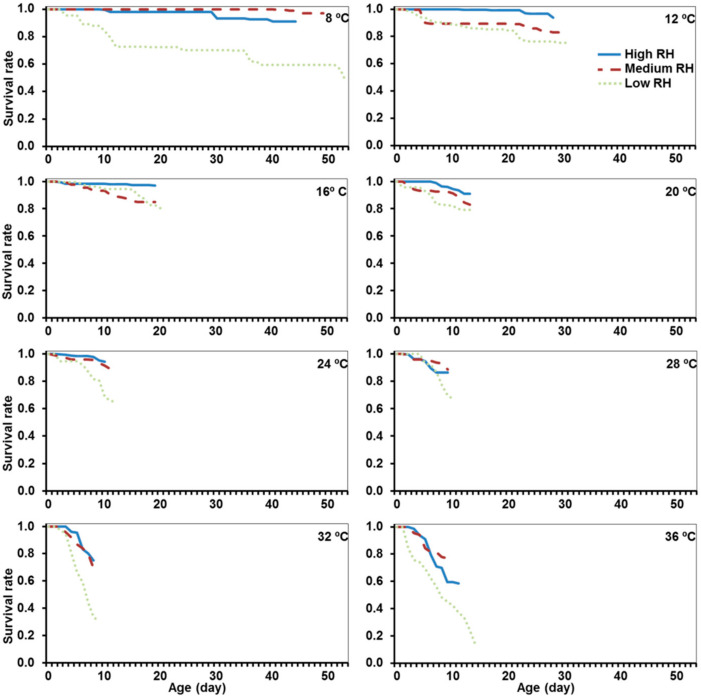
The age-specific survival rate (*lx*) of *Hypera postica* eggs at different temperatures (from 8 to 36 °C) and three relative humidity (RH) regimes (high: 90% < RH < 100%; medium: 50% < RH < 75%; and low: 10% < RH < 35%). The blue line refers to the high humidity regime; the dashed red line to the medium humidity regime, and the light green dashed line to the low humidity regime.

Eggs of the alfalfa weevil successfully developed until larval emergence at any of the eight temperatures and three RH tested. However, the temperature and RH affected the survival of the eggs. The age-stage survival rates (*lx*) are shown in Figure 1. Overall, the survival curves were significantly different (χ^2^ = 2084, df = 23; *p* < 0.001), as well as the survival curves of eggs reared at different temperatures within each RH regime (χ^2^ = 378 for high RH, χ^2^ = 204 for medium RH and χ^2^ = 758 for low RH, df = 7, and *p* < 0.001 for all relative humidity regimes). However, the RH affected the survival within each temperature (Table 1). 

Three different patterns of the effect of RH could be determined: (1) at 8 and 36 °C, differences between the three RH regimes occurred, and the highest survival was recorded at the medium RH regime. (2) From 12 to 20 °C, the highest survival was recorded at high RH, and there were no significant differences between the medium and low RH regimes. (3) The last pattern occurred at intermediate-high temperatures (24 to 32 °C), when the lowest survival was associated with low RH, and there were no differences between medium and high RH regimes (Figure 1 and Table 1).

### 3.2. Developmental Time

**Figure 2:**
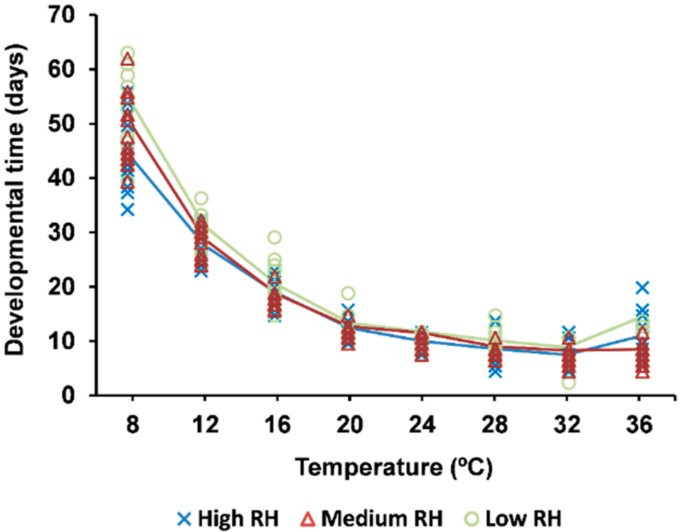
The egg developmental time of *Hypera postica* at different temperatures and relative humidity (RH) conditions (high: 90% < RH < 100%; medium: 50% < RH < 75%; and low: 10% < RH < 35%).

Temperature significantly affected the duration of the development (χ^2^ = 3566.2; *p* < 0.0001). The incubation period of *H. postica* eggs (Figure 2) decreased when the temperature increased from 8 to 32 °C but increased again at 36 °C. Differences between the RH regimes occurred (χ^2^ = 29.4; *p* < 0.0001) with the fastest development occurring at high RH and the slowest at low RH. No significant interaction between the temperature and RH was found (χ^2^ = 18.1; *p* = 0.2027).

### 3.3. External Egg Colour

**Figure 3:**
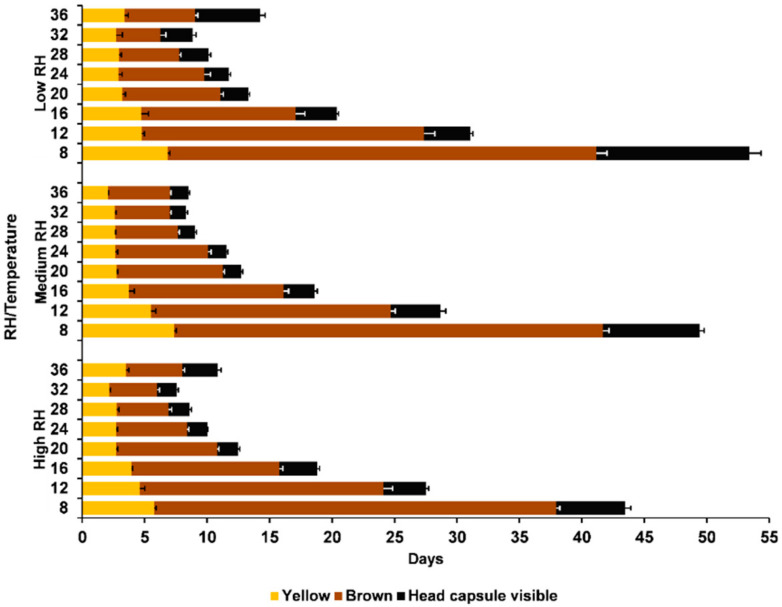
The external egg colour stages of the alfalfa weevil, *Hypera postica*, at different temperatures and relative humidity regimes (high: 90% < RH < 100%; medium: 50% < RH < 75%; and Low: 10% < RH < 35%). Yellow: early developmental stage when the egg was yellow; Brown: intermedium developmental stage when the egg was brown; and Black: end of the developmental stage when the head capsule was visible.

There were significant differences in the duration of the egg colour stages (χ^2^ = 2720.38; *p* < 0.0001) (Figure 3). These differences were also significantly affected by the temperature (χ^2^ = 2972.49; *p* < 0.0001) and RH (χ^2^ = 27.14; *p* < 0.0001). However, the duration of the egg colour stages was also significantly affected by the interaction with the RH (χ^2^ = 37.30; *p* < 0.0001). The eggs spent more time in the brown stage than in the other two stages (Figure 3). At low RH, the yellow stage was significantly shorter than the head capsule visible stage, whereas at medium and high temperatures, it was the contrary.

### 3.4. Developmental Rate

**Figure 4:**
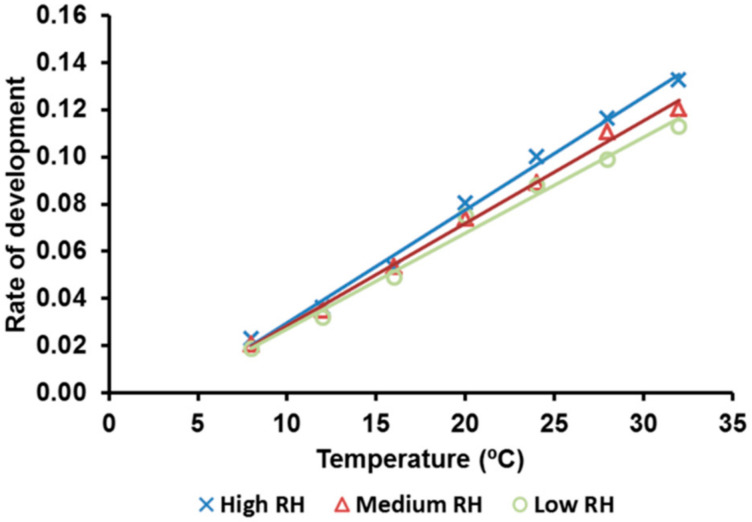
The linear relationship between temperature and the rate of development of the alfalfa weevil eggs, *H. postica*, at different temperatures and relative humidity (high: 90% < RH < 100%; medium: 50% < RH < 75%; and low: 10% < RH < 35%).

When the rates of the development of eggs were fitted against the temperatures tested, the relationship was adequately described by the linear model for any of the three RH regimes (Figure 4, Table 2). The minimum developmental threshold (T_0_) varied according to the HR regime. However, an inverse relationship was found for the thermal requirement (K) for completing the egg development (Table 2).

### 3.5. Oviposition Window in the Ebro-Valley

Based on the T_0_ and K, which were previously calculated with the linear model for each RH regime (Table 2), the weekly theoretical hatching periods (with the thermal requirements) according to the potential oviposition date are shown in Supplementary Table S1 for each RH regime and area of the Ebro Valley. Eggs laid during the first week of October will hatch two weeks later at any RH regime. Slight variations in the hatching time between areas and RH regimes will occur if the eggs are laid in other weeks of October. Some differences in the forecasted hatching time could occur between areas and RH regimes with eggs laid from November in advance. However, the usual field climatic conditions in autumn and winter coincide with the medium RH values, and this was used to determine the oviposition window.

**Figure 5:**
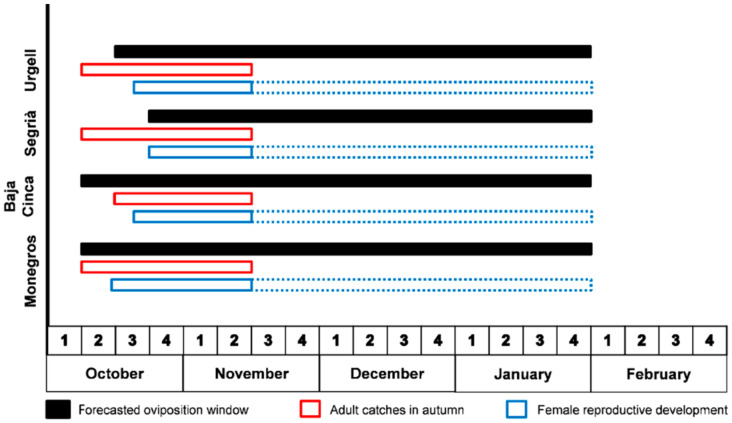
The oviposition windows of *Hypera postica* for the four areas of the Ebro Valley at the medium relative humidity regime (50% < RH < 75%). Black rectangle: the forecasted oviposition window; Red rectangle: the period with adult catches in autumn; and Blue rectangle: the period with females having mature eggs in the reproductive system/ovarioles. The dashed blue rectangle indicates that oviposition likely continued, although no sampling was made after the third week of November.

The theoretical range of the oviposition period, shown in Supplementary Table S1, could be adjusted to the actual oviposition window with (1) the field records of the larvae and eggs (Supplementary Table S2) and (2) the occurrence of adults in the autumn and the status of their reproductive systems (Supplementary Table S3). In fact, the theoretical prediction showed that the beginning of the oviposition window varied according to the area of the Ebro Valley and ranged from the second to the fourth week of October. However, the first reproductive adults were mainly caught after the second week of October in all four areas of the region (Supplementary Table S3). Therefore, the oviposition windows varied very little between areas (Figure 5). 

## 4. Discussion

Several authors have studied the effects of temperature on the alfalfa weevil development [14,18,30,31]. However, few observed the combined effect of the relative humidity and temperature during embryonic development. Our study shows that both abiotic factors affected the survival and the embryonic development.

We found that *H. postica* eggs were capable of surviving and developing across a wide range of temperatures from 8 to 36 °C. These results mainly agree with the works of other authors [14,31], who tested 15 and 11 temperatures, respectively, within the range of 6–37 °C. However, no egg development at 9 and 37 °C was found [14], whereas, in our experiment, nearly the totality of the eggs survived and developed well at 8 °C, and a noticeable proportion did so at 36 °C as with [31], who reported that, at these limits, only 42% and 57% survived, respectively. They also reported the apparent development at 6 °C after 60 days of incubation, but only those that were transferred to higher temperatures, after this period, hatched and survived. 

The reasons for those differences at extreme temperatures could be due to the RH at which the experiments were performed. In our experiment, egg mortality was much greater at low RH than at medium or high RH levels. Although, at low temperatures, very few eggs of *H. postica* hatched at humidity levels below 95% in a previous study [30], our results showed that nearly 50% of the eggs survived at a low RH regime. The differences between our results and others from North America or Middle East could be due to differences between regional populations [19,21], as has been reported when the influence of temperature in the postembryonic development of *H. postica* was studied [14,18].

Overall, the higher the temperature, the lower the rate of egg survival and this drastically dropped at 32 and 36 °C. When temperature was combined with dry environments, a low percentage of embryos completed development. We observed that, in most cases, the fully-developed larva struggled for several days to hatch, ending up dead without making it out of the egg. A low RH level has often been recorded as interfering with the egg hatching process [32]. *Dinoderus minutus* (Coleoptera: Bostrichidae had great difficulty in hatching at low humidity, and many larvae were unable to extricate themselves from the egg [33]. 

Our results revealed that the RH did not have an equal affect at all temperatures: (1) at extreme temperatures (8 and 36 °C), the highest survival was at the medium RH. It is difficult to explain this feature at 8 °C. However, researchers reported that the rove beetle *Ocypus olens* (Coleoptera: Staphylinidae) survived only 3–4 days at low temperatures and high RH [34], as this affected the egg plastron. The negative interaction of high HR and high temperatures is better known for affecting the development of the embryo [15,33,35,36,37]. (2) Between 12 and 20 °C, the highest survival was at a high RH regime. The positive effect of a high HR on *H. postica* egg hatching was previously reported [30]. However, our results differ from those of these authors because a high proportion of eggs hatched even at a medium and low HR. (3) The survival of eggs was higher at a medium RH at middle-high temperatures (24, 28, and 32 °C), indicating that a globally medium RH is the best regime for egg hatching for Spanish populations of *H. postica,* which was also observed in studies done with populations from the Asian Middle East [14,19].

The egg developmental time was affected by the temperature and RH, but no interaction between these two factors occurred. As expected, the higher the temperature, the shorter the hatching time in the range of 8–32 °C. These results agree with previous studies [18,30,31]. On the other hand, the hatching time was longer at low rather than at medium and high RH regimes as was previously stated for other insects [15,38]. An increase in the egg developmental time was observed when the RH was lower than 70% [30]. In some cases, at low RH environments, the extension of the development appeared to be a result of the depression of the egg metabolism due to water loss [39]. 

The results of the present study indicate that, at all temperature-RH combinations tested, the brown colour was the categorical stage where the eggs spent more time. These results agree with several authors [22,23,40]. The alfalfa weevil eggs were very susceptible to low temperatures at the beginning and at the end of the embryonic stage [41]. However, they became increasingly tolerant to cold temperatures as the egg reached the brown category [40]. Related to the susceptibility to low temperatures at the head capsule stage, another study [42] showed that this can be due to desiccation of the chorion and hindering of the hatching.

The temperature-dependent development rates fit well to the linear model, and T_0_ and K could be used for predicting a theoretical oviposition window. For that purpose, we used only medium RH because the usual climatic condition in autumn and winter coincided with these values. The field samplings made in autumn (adults) and winter (eggs and larvae) allowed us to more accurately determine the beginning and the end of this oviposition window. The window lasts from the third week of October to the end of January, with only small differences between the areas of the Ebro Valley. This result is not surprising considering that there was a straight-line distance between areas of about 150 km and no remarkable differences in latitude and altitude between those areas.

The oviposition window allows a partial explanation of the success of *H. postica* in the region. Adults enter the field after passing through summer diapause (Table S3). Somehow, these results are in concordance with other authors [3]. Although, in October or November, alfalfa enters the vegetative-stop period, the stems remain green and turgid, perfect for female egg laying. The temperatures are usually widely above the eggs developmental threshold (estimated at 3.4 °C), the RH inside the stems is high, and low egg mortality could be expected [40,43]. During winter, after the first frosts, the plants become completely dried. However, the foggy weather, typical in the region, and mean temperatures around 8 °C provide sufficiently suitable environmental conditions to ensure high survival. On the other hand, the first larvae were already recorded in the second fortnight of November in certain fields and years. This indicates that a proportion of the population of the alfalfa weevil will spend the winter as larvae, and the potential damage will start soon after alfalfa dormancy period ends, also stated by several authors in other counties [44,45].

However, global warming can enhance the potential of damage of *H. postica* in the studied region and in other regions of the Mediterranean Basin. The Mediterranean is one of the world’s regions where a 20% faster increase in temperature has been forecasted compared to other regions of the world. The mean temperature in the northeast of the Iberian Peninsula has increased by 1.2 °C in the last 65 years [46], and temperatures are predicted to be 2.2 °C above the average in 2040 [47]. Within this new framework, the egg development will be faster and the potential lower RH conditions will not be enough to reduce the egg survival and to increase the developmental time. In any case, control methods that prevent or hinder the egg survival and development before the start of the alfalfa vegetative season would be a good tool for reducing the pest incidence. Winter grazing and alfalfa cutting in late autumn could reduce substantially the overwintering *H. postica* populations. The efficacy of both methods has been reported in North America [45,48]. However, grazing is in disuse in Spain and the efficacy of a winter cutting in the Mediterranean crop conditions could be a useful tool to prevent damages at the beginning of the alfalfa vegetative season. Studies in this sense are being developed by our research group.

Our results provide important information on the egg development and survival of the alfalfa weevil under constant temperatures and different relative humidity regimes, which should be exploited in integrated pest management strategies. However, to better understand the phenology and develop a more precise management strategy that is feasible for decision making, knowledge regarding how environmental abiotic factors affect the larval stage is necessary. We are developing an experiment in this sense, and our provisional data indicate that the mortality at 8 and 12 °C is very low [29]. 

## 5. Conclusions

The results of the study showed that the temperature and relative humidity are important factors in the survival and development time of Spanish populations of *H. postica*. The lowest egg survival occurred at high temperatures and low relative humidity. The egg developmental time decreased from 8 to 32 °C, and, at a low RH, this time was longer. The minimum development threshold (T_0_) and the thermal requirement for egg development (K) ranged between 3 and 4 °C and 209 and 246 degree-days, respectively. According to these values and the occurrence of eggs and larvae (in winter) and adult (in autumn) in field samplings, the period of oviposition was determined, which started from the second week of October with small differences between the areas of the studied region. The results of the study contribute to a better understanding of the annual cycle and phenology of *H. postica* and are useful for developing sustainable pest management strategies, for example grazing or winter cutting during the alfalfa dormancy period, which can hinder the egg survival and development before starting alfalfa vegetative season. 

## Figures and Tables

**Table 1:** Log-Rank test comparing age-specific survival rate (*lx*) curves of *Hypera postica* eggs at three RH regimes (high: 90% < RH < 100%; medium: 50% < RH < 75%; and low: 10% < RH < 35%) at eight temperatures (from 8 to 36 °C).

RH Regimes	8 °C			12 °C			16 °C			20 °C			24 °C			28 °C			32 °C			36 °C		
	χ^2^	df	*p*	χ^2^	df	*p*	χ^2^	df	*p*	χ^2^	df	*p*	χ^2^	df	*p*	χ^2^	df	*p*	χ^2^	df	*p*	χ^2^	df	*p*
3 RH	174	2	<2 × 10^−16^	29.7	2	4 × 10^−7^	26.3	2	2.2 × 10^−6^	14.7	2	7 × 10^−4^	63.6	2	2 × 10^−14^	31.1	2	2 × 10^−7^	134		<2 × 10^−16^	62.2	2	3 × 10^−14^
High vs. medium	20.4	1	6 × 10^−6^	13.4	1	3 × 10^−4^	19.6	1	1 × 10^−5^	7.3	1	0.007	1.5	1	0.2	0.7	1	0.4	2	1	0.2	11.4	1	7 × 10^−4^
High vs. low	74.2	1	<2 × 10^−16^	29.8	1	5 × 10^−8^	26.7	1	2 × 10^−7^	14.8	1	1 × 10^−4^	49.6	1	2 × 10^−12^	15.6	1	8 × 10^−5^	88.4	1	<2 × 10^−16^	24.3	1	8 × 10^−7^
Medium vs. low	129	1	<2 × 10^−16^	4.0	1	0.06	0.2	1	0.7	1.1	1	0.3	28.7	1	8 × 10^−8^	25.6	1	4 × 10^−7^	83.1	1	<2 × 10^−16^	47.7	1	5 × 10^−12^

**Table 2:** The linear regression parameters and the calculated values of the minimum development threshold (T_0_) and thermal constant (K) for *Hypera postica* eggs.

RH	Parameters of the Linear Regression					T_0_ (°C)	K (DD)
	Coefficient (b)	Intercept (a)	*F*	*p*	R^2^		
High	0.0048	−0.0183	757.4	<0.0001	0.9934	3.82	209.05
Medium	0.0043	−0.0147	1019.3	<0.0001	0.9907	3.40	230.83
Low	0.0041	−0.0141	373.3	<0.0001	0.9868	3.30	246.14

## Data Availability

The data presented in this study are available on request from the corresponding author.

## Supplementary Materials

The following are available online at https://www.mdpi.com/2075-4450/12/3/250/s1. Table S1: The first *Hypera postica* oviposition time and egg hatching predicted dates according to the required degree-days accumulation (K), at the three different relative humidity regimes (high: 90% < RH < 100%; medium: 50% < RH < 75%; and low: 10% < RH < 35%), considering the temperature of the last fifteen years in four different areas of the Ebro Valley (Urgell, Segrià, Baja Cinca and Monegros). Table S2: The percentages of the egg colour stages (yellow, brown, and head capsule visible (HCV)) and larvae stages of *Hypera postica,* recorded in the four areas of the Ebro Valley (November until February) from the 2019–2020 crop seasons. Table S3: The number of collected and mature males and females of *Hypera postica* adults from sweep net sampling over 6 weeks, from the beginning of October until mid-November of the 2019 and 2020 crop seasons. Figure S1. Scheme of the link between laboratory experiments and field sampling records. Grey bars means the sampling periods of *Hypera postica* during the alfalfa dormancy period between October and February in the Ebro Valley (Spain). Red arrows and red text mean the field records. Green dashed arrows and text mean the use of the results of the laboratory experiments at medium RH to forecast and determine egg laying events K, degree-days needed by eggs to complete development; Td L1, developmental time needed for completing the 1st instar larvae at 8 °C obtained from [29]; Td YE, developmental time for yellow eggs at 8 °C.
