# Supplementary material for: Effects of Temperature and Relative Humidity on the Embryonic Development of Hypera postica Gyllenhal (Col.: Curculionidae)
Source: Insects. 2021 Mar 16;12(3):250. doi: 10.3390/insects12030250 (PMC8001389; doi:10.3390/insects12030250)
Supplement: Supplementary file 1 [file insects-12-00250-s001.pdf]

Table S1. The first *Hypera postica* oviposition time and egg hatching predicted dates according to the required degree-days accumulation (K), at the three different relative humidity regimes (high: 90% < RH < 100%; medium: 50% < RH < 75%; and low: 10% < RH < 35%), considering the temperature of the last fifteen years in four different areas of the Ebro Valley (Urgell, Segrià, Baja Cinca and Monegros).

Potential Oviposition Date		Theoretical Hatching Period											
		Low RH				Medium RH				High RH			
**Month**	**Week**	**URGELL**	**SEGRIÀ**	**BAJO CINCA**	**MONEGROS**	**URGELL**	**SEGRIÀ**	**BAJO CINCA**	**MONEGROS**	**URGELL**	**SEGRIÀ**	**BAJO CINCA**	**MONEGROS**
**Oct**	1	Oct 16-23	Oct 16-23	Oct 16-23	Oct 16-23	Oct 16-23	Oct 16-23	Oct 16-23	Oct 16-23	Oct 16-23	Oct 16-23	Oct 16-23	Oct 16-23
	2	Oct 23-30	Oct 23-31	Oct 23-31	Oct 23-31	Oct 23-30	Oct 23-31	Oct 23-31	Oct 23-31	Oct 23-30	Oct 23-30	Oct 23-31	Oct 23-31
	3	Nov 7-16	Nov 1-7	Nov 7-16	Nov 1-7	Nov 7-16	Nov 1-7	Nov 1-7	Nov 1-7	Nov 1-7	Nov 1-7	Nov 1-7	Nov 1-7
	4	Nov 23-30	Nov 16-23	Nov 23-30	Nov 16-23	Nov 23-30	Nov 16-23	Nov 23-30	Nov 16-23	Nov 23-30	Nov 16-23	Nov 16-23	Nov 16-23
**Nov**	1	Jan 7-16	Dec 7-16	Jan 16-23	Dec 7-16	Jan 1-7	Dec 1-7	Jan 1-7	Dec 1-7	Jan 1-7	Dec 7-16	Jan 16-23	Dec 1-7
	2	Feb 1-7	Jan 1-7	Feb 7-16	Jan 1-7	Jan 16-23	Dec 23-31	Feb 1-7	Dec 23-31	Feb 1-7	Jan 1-7	Feb 7-16	Dec 23-31
	3	Feb 16-23	Jan 23-31	Feb 23-28	Jan 16-23	Feb 7-16	Jan 16-23	Feb 16-23	Jan 7-16	Feb 16-23	Jan 23-31	Feb 23-28	Jan 16-23
	4	Mar 1-7	Feb 7-16	Mar 1-7	Feb 1-7	Feb 23-28	Feb 1-7	Mar 1-7	Feb 1-7	Feb 23-28	Feb 7- 16	Mar 1-7	Feb 1-7
**Dec**	1	Mar 7-16	Feb 16-23	Mar 7-16	Feb 16-23	Mar 1-7	Feb 16-23	Mar 7-16	Feb 7-16	Mar 1-7	Feb 23-28	Mar 7-16	Feb 16-23
	2	Mar 7-16	Feb 23-28	Mar 7-16	Feb 16-23	Mar 1-7	Feb 16-23	Mar 7-16	Feb 16-23	Mar 7-16	Feb 23-28	Mar 7-16	Feb 16-23
	3	Mar 7-16	Mar 1-7	Mar 7-16	Feb 23-28	Mar 1-7	Feb 23-28	Mar 7-16	Feb 23-28	Mar 7-16	Mar 1-7	Mar 7-16	Feb 23-28
	4	Mar 7-16	Mar 1-7	Mar 16-23	Mar 1-7	Mar 7-16	Mar 1-7	Mar 7-16	Feb 23-28	Mar 7-16	Mar 1-7	Mar 7-16	Mar 1-7
**Jan**	1	Mar 7-16	Mar 1-7	Mar 16-23	Mar 1-7	Mar 7-16	Mar 1-7	Mar 7-16	Mar 1-7	Mar 7-16	Mar 1-7	Mar 16-23	Mar 1-7
	2	Mar 16-23	Mar 1-7	Mar 16-23	Mar 1-7	Mar 7-16	Mar 1-7	Mar 16-23	Mar 1-7	Mar 7-16	Mar 1-7	Mar 16-23	Mar 1-7
	3	Mar 16-23	Mar 7-16	Mar 16-23	Mar 7-16	Mar 7-16	Mar 1-7	Mar 16-23	Mar 1-7	Mar 16-23	Mar 7-16	Mar 16-23	Mar 1-7
	4	Mar 16-23	Mar 7-16	Mar 16-23	Mar 7-16	Mar 16-23	Mar 7-16	Mar 16-23	Mar 7-16	Mar 16-23	Mar 7-16	Mar 16-23	Mar 7-16
**Feb**	1	Mar 16-23	Mar 7-16	Mar 23-31	Mar 7-16	Mar 16-23	Mar 7-16	Mar 16-23	Mar 7-16	Mar 16-23	Mar 7-16	Mar 16-23	Mar 7-16
	2	Mar 23-31	Mar 16-23	Mar 23-31	Mar 16-23	Mar 16-23	Mar 16-23	Mar 23-31	Mar 16-23	Mar 16-23	Mar 16-23	Mar 23-31	Mar 16-23
	3	Mar 23-31	Mar 16-23	Mar 23-31	Mar 16-23	Mar 23-31	Mar 16-23	Mar 23-31	Mar 16-23	Mar 23-31	Mar 16-23	Mar 23-31	Mar 16-23
	4	Apr 1-7	Mar 23-31	Apr 1-7	Mar 23-31	Mar 23-31	Mar 23-31	Mar 23-31	Mar 23-31	Mar 23-31	Mar 23-31	Mar 23-31	Mar 23-31

Table S2. The percentages of the egg colour stages (yellow, brown, and head capsule visible (HCV)) and larvae stages of *Hypera postica,* recorded in the four areas of the Ebro Valley (November until February) from the 2019–2020 crop seasons.

Zone	Field	Sampling Date	Egg			Larvae		
			% Yellow	% Brown	% HCV	L1	L2	L3
**2018/2019**								
SEGRIÀ (1)	1.1	22-11	21	77	2	26	0	0
	1.2	4-12	16	78	5	0	0	0
	1.3	11-12	100	0	0	0	0	0
	1.4	19-12	10	86	4	0	0	0
	1.5	19-12	3	97	0	0	0	0
	1.6	3-1	16	84	0	3	0	0
	1.7	3-1	47	41	13	3	0	0
	1.8	8-1	16	77	8	2	0	0
URGELL (2)	2.1	18-1	0	100	0	1	0	0
	2.2	18-1	0	100	0	22	0	0
	2.3	20-1	0	100	0	3	0	0
	2.4	24-1	0	75	25	45	0	0
	2.5	24-1	0	51	49	7	0	0
	2.6	25-1	5	95	0	0	0	0
	2.7	30-1	0	95	5	18	0	0
BAJA CINCA (3)	3.1	22-1	0	69	31	25	0	0
	3.2	22-1	1	67	32	78	0	0
	3.3	22-1	6	62	32	36	0	0
MONEGROS (4)	4.1	21-1	0	66	34	60	1	0
	4.2	21-1	2	57	41	73	0	0
	4.3	21-1	3	80	17	84	2	0
	4.4	21-1	2	98	0	17	0	0
	4.5	21-1	17	61	23	41	0	0
	4.6	21-1	4	89	6	81	0	0
**2019/2020**								
SEGRIÀ (1)	1.6	27-11	21	79	0	12	0	0
	1.8	17-1	0	99	1	0	0	0
	1.7	4-2	0	83	17	11	0	0
	1.3	4-2	35	58	7	14	3	1
	1.4	4-2	3	97	0	0	0	0
	1.5	4-2	0	96	4	19	0	5
URGELL (2)	2.6	10-12	8	92	0	0	0	0
	2.8	10-1	1	99	0	7	0	0
	2.9	10-1	6	92	2	11	0	0
	2.10	17-1	4	91	5	0	0	0
	2.11	25-1	0	100	0	7	11	3
	2.1	31-1	11	84	5	31	0	0
	2.2	31-1	0	0	0	1	0	0
	2.12	6-2	24	76	0	3	1	0
	2.13	6-2	0	100	0	1	0	1
	3.1	15-11	44	56	0	0	0	0

	3.3	15-11	19	81	0	0	2	0
BAJA CINCA (3)	3.1	7-2	0	100	0	19	8	4
	3.2	7-2	33	59	8	2	5	1
	3.3	7-2	19	81	0	0	2	2
MONEGROS (4)	4.6	8-11	51	41	8	5	4	0
	4.5	14-1	8	88	4	61	0	0
	4.7	7-2	5	92	3	1	0	0

Table S3. The number of collected and mature males and females of *Hypera postica* adults from sweep net sampling over 6 weeks, from the beginning of October until mid-November of the 2019 and 2020 crop seasons.

**ZONE**	**Sampling**		**Nº Collected Adults**		**Nº Mature Adults**	
	**Month**	**Week**	♂	♀	♂	♀
Urgell	**Oct**	1	0	0	0	0
		2	0	0	0	0
		3	1	4	1	3
		4	7	1	7	1
	**Nov**	1	5	3	5	3
		2	5	6	5	6
Segrià	**Oct**	1	1	0	1	0
		2	0	0	0	0
		3	0	1	0	1
		4	1	2	1	2
	**Nov**	1	3	3	3	3
		2	8	14	8	13
Baja Cinca	**Oct**	1	0	0	0	0
		2	0	0	0	0
		3	7	7	7	6
		4	4	4	4	4
	**Nov**	1	9	10	9	9
		2	5	13	5	13
Monegros	**Oct**	1	7	0	7	0
		2	5	7	5	6
		3	20	17	20	15
		4	5	21	5	13
	**Nov**	1	6	11	4	10
		2	3	7	3	7

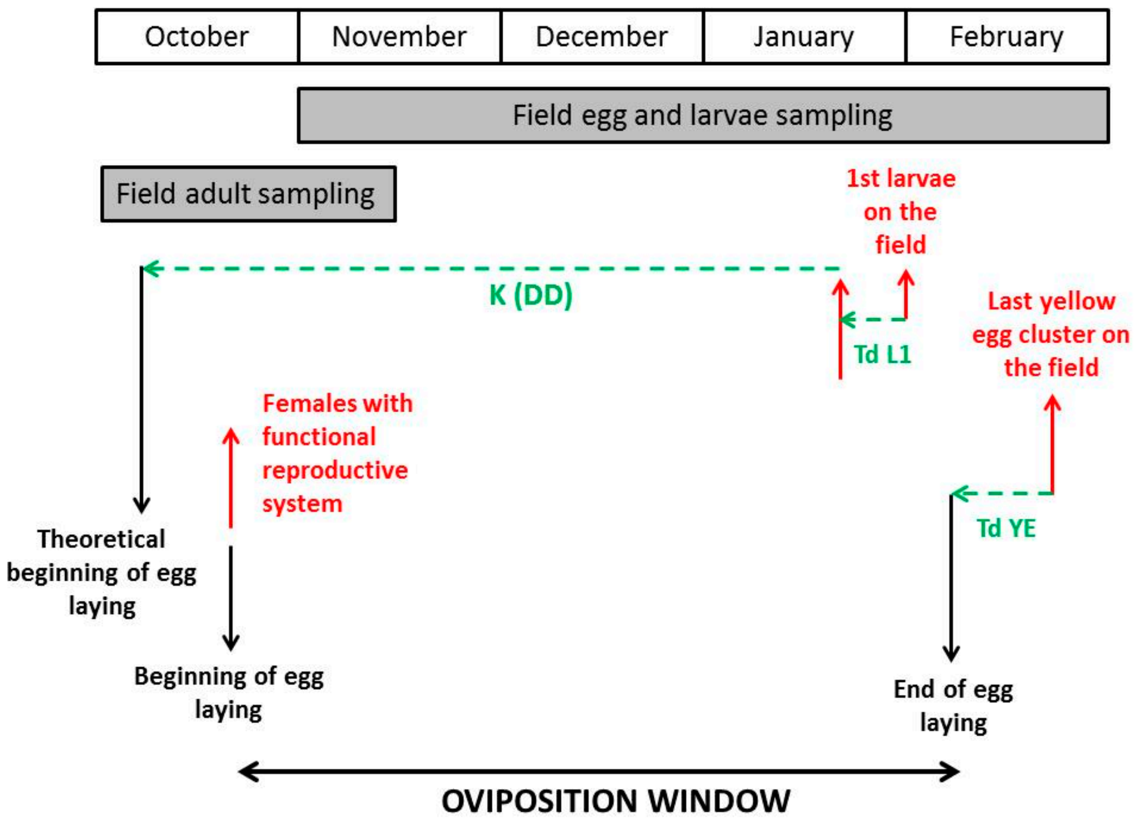

Figure S1. Scheme of the link between laboratory experiments and field sampling records. Grey bars means the sampling periods of *Hypera postica* during the alfalfa dormancy period between October and February in the Ebro Valley (Spain). Red arrows and red text mean the field records. Green dashed arrows and text mean the use of the results of the laboratory experiments at medium RH to forecast and determine egg laying events (K, degree-days needed by eggs to complete development; Td L1, developmental time needed for completing the 1st instar larvae at 8°C (obtained from [1]; Td YE, developmental time for yellow eggs at 8°C.

Reference

1. Levi-Mourao, A.; Meseguer, R.; Pons, X. Effect of temperature on the post-embryonic development and reproduction of the alfalfa weevil, *Hypera postica* Gyllenhal (Col.: Curculionidae). Status (unpublished; manuscript in preparation).
